# Historical spatial range expansion and a very recent bottleneck of *Cinnamomum kanehirae *Hay. (Lauraceae) in Taiwan inferred from nuclear genes

**DOI:** 10.1186/1471-2148-10-124

**Published:** 2010-04-30

**Authors:** Pei-Chun Liao, Dai-Chang Kuo, Chia-Chia Lin, Kuo-Chieh Ho, Tsan-Piao Lin, Shih-Ying Hwang

**Affiliations:** 1 Department of Life Science, Pingtung University of Science and Technology, Pingtung 91201, Taiwan; 2 Institute of Plant Biology, National Taiwan University, Taipei 10617, Taiwan; 3 Department of Life Science, National Taiwan Normal University, Taipei, 11677, Taiwan; 4 Department of Life Sciences, National Taiwan University, Taipei 10617, Taiwan

## Abstract

**Background:**

Species in the varied geographic topology of Taiwan underwent obvious demographic changes during glacial periods. *Cinnamomum kanehirae *has been exploited for timber and to obtain medicinal fungi for the past 100 years. Understanding anthropogenic factors influencing the demography of this species after the last glacial maximum (LGM) is critically important for the conservation of this species.

**Results:**

Populations of *C. kanehirae *were classified into four geographic regions: northwestern (NW), west-central (WC), southwestern (SW), and southeastern (SE). In total, 113 individuals from 19 localities were sampled, and variations in the chalcone synthase gene (*Chs*) intron and leafy (*Lfy*) intron-2 sequences of nuclear DNA were examined in order to assess phylogeographic patterns, the timescales of demographic and evolutionary events, and recent anthropogenic effects. In total, 210 *Chs *and 170 *Lfy *sequences, which respectively constituted 36 and 35 haplotypes, were used for the analyses. Estimates of the migration rate (*M*) through time revealed a pattern of frequent gene flow during previous and the present interglacials. The isolation-by-distance test showed that there generally was no significant correlation between genetic and geographic distances. The level of among-region genetic differentiation was significant when comparing eastern to western populations. However, no significant among-region genetic differentiation was found in comparisons among the four geographic regions. Moreover, essentially no genetic structuring was found for the three regions west of the CMR. A fit of spatial range expansion was found for pooled and regional samples according to the non-significant values of the sum of squared deviations. Using the Bayesian skyline plot (BSP) method, a recent bottleneck after the LGM expansion was detected in both regional and pooled samples.

**Conclusions:**

Common haplotype distributions among geographic regions and the relatively shallow genetic structuring displayed are the result of historical gene flows. Southward dispersals in an earlier time frame from the NW region and in a later time frame from the SE region were inferred. The BSP analysis suggested a postglacial expansion event. Recent trends, however, refer to a bottleneck due to human interventions observed for both pooled and regional *C. kanehirae *samples.

## Background

Climate change is an important factor influencing organisms' evolutionary history, especially their demographic dynamics [1]. The Pleistocene was an era of temperature oscillations due to cyclic glacial/interglacial events since 0.9 million years ago (Mya) [2]. Many species experienced huge bottleneck events during glacial periods because their growth under the prevailing unpleasant cold and dry conditions was restricted to refugia [1,2]. Contraction into refugia was reported for both animals [3-5] and plants [6-8]. Although glacial events occur on a global scale, the lowlands of southeastern Asia were not covered with ice but were drier and colder. Thus, the climatic changes were cyclic pluvial and interpluvial in Asia (respectively corresponding to interglacial and glacial periods) during the Pleistocene. According to Hewitt's [2] speculation on the climate of tropical mountains according to several faunal studies, stable moist habitats in areas not covered by ice provided suitable refugia for species survival and the generation of new lineages. Glacials changed climates in Asia and several refugia of different species are consistently being found [9-12], which shows that climate changes in the Pleistocene indeed affected the demographic history of Asian species.

Taiwan is a continental island situated off the coast of southern East Asia. The Central Mountain Ridge (CMR) is a continuous ridge trending north-south and divides Taiwan into eastern and western geographic areas [13]. There are more than 200 peaks exceeding 3000 m in elevation in the CMR. The varied geographical topologies of Taiwan allowed habitats and lineages to persist through altitudinal shifts during glacial/interglacial cycles [cf. [2]]. The remnants of glaciations at the top of some peaks indicate the former occurrence of glaciers [14,15]. The floristic composition of Taiwan is characterized by high levels of endemism (40%) and species diversity [16]. Forests in middle to low elevations of Taiwan consist of evergreen broadleaf trees, including camphor (*Cinnamomum *spp.), banyan (*Ficus *spp.), beech (*Fagus *spp.), etc. During glacial periods, conifers which were distributed at high elevations in Taiwan spread to lower elevations to escape the ice-covered mountaintops [14,17,18], and those species originally distributed in middle and low elevations slowly migrated to the lowlands. However, the dry environments of the lowlands during glacial periods may have restricted the distributions of broadleaf trees and resulted in bottleneck effects. Following glacial periods, climates of interglacial periods were warm and moist in Taiwan, and populations would have recovered and expanded. Climate-dependent changes in demographics of trees in Taiwan are evidenced by the pollen record [19] and genetics, e.g., *Michelia formosana *(Magnoliaceae) [20], *Cunninghamia konishii *(Cupressaceae) [21], and *Quercus *(or *Cyclobalanopsis*) *glauca *(Fagaceae) [22].

**Figure 1:**
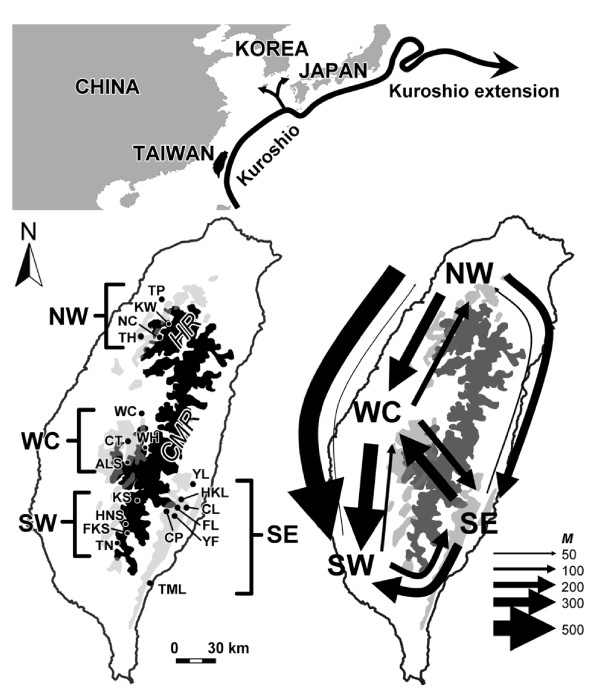
**Geographical distribution and magnitudes of migration routes of *Cinnamomum kanehirae***. In the upper panel, the flow of the Kuroshio Current is shown. In the bottom left panel, four geographic regions defined by Lin *et al*. [35] together with the location of populations are indicated. The northwestern region (NW) contains populations TP, KW, TH, and NC; the west-central region (WC) contains populations WC, CT, WH, and ALS; the southwestern region (SW) contains populations KS, HNS, FKS, and TN; and the southeastern region (SE) contains populations FL, HKL, CL, YL, CP, YF, and TML. Abbreviations of population codes are listed in Table 1. The dark area indicates elevations above 2000 m, and the gray area indicates the distribution range of *C. kanehirae *outside the area above 2000 m [26,35]. HR, Huseshan Range; CMR, Central Mountain Ridge. In the bottom right panel, directions and magnitudes of gene flow estimated by the Bayesian approach are shown. Arrows indicate the direction of migration and the thickness of the arrows indicates values of the migration parameter (*M*) calculated by the computer software, MIGRATE 3.0 [54].

In this study, we chose *Cinnamomum kanehirae *Hayata (Lauraceae) as an example to explore the influence of the evolutionary history of middle-elevation broadleaf trees. *Cinnamomum kanehirae *belongs to the *Cinnamomum *group of the Cinnamomeae *sensu *Kostermans. The *Cinnamomum *group shows an amphi-Pacific disjunct distribution with its sister group, the *Persea *group, the disruption of boreotropical ranges of which is attributed to Eocene-Oligocene climatic cooling. The genus *Cinnamomum *is considered to have originated about 35 ± 6 or 138 ± 19 Mya by estimates of the relaxed molecular clock using outgroups with two different divergence times [23]. The genus *Cinnamomum *comprises approximate 250 species distributed in tropical and subtropical eastern Asia, Australia, and the Pacific islands, and 8 of 14 *Cinnamomum *species in Taiwan are endemic, including *C. kanehirae*. *Cinnamomum kanehirae *is distributed at 200~2000 m in elevation, and is mixed with *C. micranthum *in broadleaf forests throughout the island [24]. *Cinnamomum kanehirae *has a long lifespan, was abundant at the beginning of the 20th century and is one of the most valuable timber species in Taiwan [25]. The range distribution of *C. kanehirae *is contiguous throughout Taiwan except the northeastern part east of the CMR [25,26] (Figure 1). A fungus called *Antrodia cinnamomea *Chang & Chou, parasitic inside the *C. kanehirae *trunk [27-29], is valued as a medicine due to its antioxidant [30-32], anticancer [33], antiviral [34], and antibiotic properties [33]. In total, 46,000 trees consisting of live, dead, and root stumps were recorded during an extensive survey of all areas, with the exception of most of the southeastern part of Taiwan between 1918 and 1922 [26], but it is now extremely difficult to find a standing live tree in the wild due to the intensive logging during 1920~1970 especially of populations of the northwestern and southwestern regions as well as selective cutting to obtain the medicinal fungus in recent years.

According to an allozyme survey, *C. kanehirae *has a relatively higher genetic diversity in terms of heterozygosity and other parameters compared to those of other local tree species [35]. DNA is a more-sensitive genetic marker, and is very useful for studying the demographic history and/or migratory footprints of species. Phylogeographic studies of several species in Taiwan consistently point to a rapid postglacial expansion event. The suggestion of population expansion of most of those studies was based on the "star-like" phylogeny of chloroplast DNA (cpDNA) haplotypes due to the high frequencies of singletons [21,36,37]. Approaches based on the coalescence theory are used to trace the evolutionary history, e.g., the Bayesian skyline plot (BSP) analysis [38]. Plastid DNA is commonly used for making phylogeographic inferences; however, a strong selective sweep acting across the entire plastid genome can reduce the genetic variation of plastid DNA [cf. [39]]. The genetic variation of *C. kanehirae *estimated from cpDNA is very low (only six haplotypes in populations and *π *= 0.00016 ± 0.00007) [40], and the amounts of variation cannot reflect the long-term demographic history or the historical effective population size. Nuclear DNA is a suitable marker to obtain more information so that the evolutionary history of *C. kanehirae *can be traced in Taiwan.

In this study, the intron of chalcone synthase (*Chs*) and the second intron of the Leafy gene (*Lfy*) of nuclear DNA were sequenced to assess the phylogeographic structure of *C. kanehirae *in Taiwan. Population genetic analyses of *C. kanehirae *should provide more insights into geographic influences on migratory events (historical gene flow and recent dispersal). Furthermore, a coherent timescale for the geologic time and demographic events as well as climate change was established using the coalescent-based Bayesian framework. The ancestral effective population size of *C. kanehirae *was estimated in order to understand the trend of historical demography. Such a study is vital to our understanding of the process of demographic changes corresponding to how climate changes affected subtropical tree species in the Pleistocene.

## Methods

### Plant materials and DNA amplification

In total, 113 individuals originating from 19 populations of *C. kanehirae *of Taiwan were collected from a scion garden at the Liukuei Research Center, Taiwan Forestry Research Institute (TFRI), southwestern Taiwan (Table 1, Figure 1). This scion garden was set up in 1995 to house intraspecific variations of *C. kanehirae*. Sample sizes of each population range from 3 to 16 individuals. Young developed leaves were collected from this garden, preserved on silica gel, and carried back to the laboratory for DNA extraction. Total DNA was extracted from ground-up leaf powder according to a modified cetyltrimethyl ammonium bromide (CTAB) procedure [41]. DNA was precipitated with ethanol and after washing with 70% ethanol, was dissolved in 200 μL TE buffer (pH 8.0) and stored at -20°C. The DNA concentration was determined for each sample using the GeneQuant II RNA/DNA Calculator (Amersham Biosciences, Taipei, Taiwan).

**Table 1:** Sampling sites, population codes, region to which a population belongs, longitude and latitude of populations, and haplotypes in each population of *Cinnamomum kanehirae *in Taiwan.

Population	Code	Geographic regions	Elevation (m)	Longitude	Latitude	*Chs *Haplotypes	*Lfy *Haplotypes
Kuanwu	KW	Northwestern (NW)	1500	121°07'12"E	24°30'36"N	4(3), 5(2), 22(1)	1(2), 11(1), 14(1), 26(2)
Nanchuang	NC	Northwestern (NW)	1100	121°03'00"E	24°24'36"N	3(1), 4(3), 13(1), 14 (12), 15(2), 24(1)	1(10), 11(1), 14(1)
Tahu	TH	Northwestern (NW)	1400	120°52'12"E	24°25'12"N	4(1), 15(1), 22(3), 31(1)	1(4), 11(2), 14(2)
Taping	TP	Northwestern (NW)	1300	121°03'36"E	24°40'48"N	2-3(1), 4(3), 15(2), 34(3)	1(2), 3(2), 26(2), 33(1)
Alishan	ALS	West-central (WC)	2000	120°47'24"E	23°30'36"N	1-2(2), 3(1), 4(3), 5(1), 6(1)	1(2), 2(2)
Chitou	CT	West-central (WC)	1500	120°47'24"E	23°39'36"N	1(3), 3(1), 4(7), 5(2), 9(1), 12(1), 13(1), 14(4), 15(3), 16(1), 17(1), 18(1), 19(1), 20(2), 21(1)	11(1), 12(2)
Wuchieh	WC	West-central (WC)	600	120°54'36"E	23°51'36"N	3(2), 4(2), 30(1)	12(1), 17(1), 34(1), 35(1)
Wanghsiang	WH	West-central (WC)	1100	120°55'48"E	23°37'12"N	3(2), 15(1), 16(1), 35(1)	25(1), 26(5)
Fengkangshan	FKS	Southwestern (SW)	2100	120°47'24"E	23°00'00"N	1(11), 2(1), 4(4), 9(1), 22(1), 23(2), 24(1), 25(1)	1(14), 11(2), 13(1), 14(2)
Hsinanshan	HNS	Southwestern (SW)	1000	120°46'48"E	23°03'36"N	4(1), 5(1), 15(1), 27(1), 28(1)	11(4), 22(4)
Kuanshan	KS	Southwestern (SW)	1000	120°54'36"E	23°24'36"N	3(4), 4(8), 8(2), 9(5), 29(1), 30(1)	1(1), 2(1), 12(2), 17(4), 22(1), 23(1), 24(1), 25(1), 26(3), 27(1), 28(4)
Tona	TN	Southwestern (SW)	1500	120°43'12"E	22°54'36"N	4(1), 15(3), 20(1), 33(2)	22(2), 32(1)
Chulu	CL	Southeastern (SE)	200	121°18'00"E	23°21'00"N	3(3), 4(2), 7(1)	3(2), 4(1), 5(3)
Chingping	CP	Southeastern (SE)	1200	121°10'12"E	23°40'48"N	3(1), 4(12), 8(1), 9(1), 11(1)	3(3), 4(1), 5(4), 6(1), 7(1), 8(1), 9(1), 10(1)
Fuli	FL	Southeastern (SE)	600	121°11'24"E	23°10'48"N	3(2), 4(2), 11(1), 15(1)	1(2), 3(1), 15(1), 16(1)
Hsiukuluan	HKL	Southeastern (SE)	700	121°13'12"E	23°15'00"N	3(7), 4(6), 8(1), 9(1), 26(1)	3(8), 12(5), 17(1), 18(2), 19(2), 20(1), 21(1)
Taimali	TML	Southeastern (SE)	600	121°00'00"E	22°30'36"N	3(3), 4(4), 9(2), 32(1)	19(2), 29(4), 30(1), 31(1)
Yungfeng	YF	Southeastern (SE)	600	121°10'12"E	23°04'12"N	3(4), 4(4), 14(1), 36(1)	1(8)
Yuli	YL	Southeastern (SE)	1000	121°15'00"E	23°11'24"N	3(2), 4(4), 9(2)	3(1), 11(2), 12(7)

The numbers in parentheses indicates the frequency of a haplotype at a particular site. Haplotypes which are population-specific are also region-specific, and they are underlined.

### Primers, polymerase chain reaction (PCR), and sequencing

PCRs were performed with universal primers for the *Chs *first intron (CHSx1F and CHSx2RN) [42] and the *Lfy *second intron (Mk.LFY-1n and Mk.LFY-2n) [43]. Amplifications were performed in a DNA programmable thermal cycler (PTC-100, MJ Research, Watertown, MA, USA) under the following protocol: initial denaturation at 94°C for 2 min followed by 35 cycles of 30 s at 94°C, 30 s of annealing at 52 or 48°C (*Chs *and *Lfy*, respectively), and 2 min at 72°C, followed by a subsequent 7-min final extension at 72°C. The PCR mixture (50 μL) contained 50 mM KCl, 1.5 mM MgCl_2_, 0.001% gelatin, 10 mM Tris-HCl (pH 8.3), 100 μM dNTPs, 0.2 μM of the primer, 20 ng of template DNA, 1 μg RNase, and 0.5 U *Taq *polymerase (Amersham Biosciences). Amplification products were run on 1% agarose gels, purified with a QiaGen purification kit (Taipei, Taiwan), and sequenced using both forward and reverse reactions with a *Taq *BigDye Terminator Cycle Sequencing Kit and a model ABI373A automated sequencer (Applied Biosystems, Foster City, CA, USA). This was applied to sequences of homozygotes or sequences containing one polymorphic site. Heterozygotes were visualized as "double peaks" at polymorphic sites in the chromatogram. The identity of the two haplotypes within a heterozygote was inferred through haplotype subtraction [44], which deduces haplotypes by a comparison of heterozygote sequences to haplotypes commonly observed in the global sample. When the chromatogram quality did not permit this procedure, PCR products were cloned using a yT&A vector (Yeastern Biotech, Taipei, Taiwan) according to the manufacturer's protocol. Three clones were sequenced for each individual when sequences contained two or more polymorphic sites and/or an unpaired indel (an indel of a different length). The two sequences of a heterozygote were separated by comparing sequences of the PCR product and cloned sequence. Fortunately, no sequence containing more than one indel was found in the length of *Chs *and *Lfy *used in this study. Because the *Taq *polymerase error was estimated to be as high as 0.1% [45] or even higher, sequences containing only one polymorphic site were considered to be produced by a *Taq *error and were excluded from the analysis. All haplotype sequences were deposited in the NCBI nucleotide sequence database under the following accession numbers: FJ858218~FJ858253 (*Chs*) and GQ260573~GQ260607 (*Lfy*).

### DNA sequence analyses

Sequence alignments were performed with Clustal X [46] and manually edited using BioEdit vers. 7.0.9 [47]. After sequence alignment, indels were treated as the fifth character when calculating the indices of genetic diversity. The nucleotide diversity (*π*), haplotype diversity (*Hd*), and *θ *estimated by segregating sites (*S*) were calculated using DnaSP vers. 5.0 [48].

### Population structure

Populations of *C. kanehirae *were formerly classified into four geographic regions, northwestern (NW), west-central (WC), southwestern (SW), and southeastern (SE), according to their geographic distributions (Figure 1) [35]. In addition, the CMR is considered to be a barrier to gene flow, and the structures of populations distributed in eastern and western Taiwan were also examined. The haplotype network was reconstructed with the algorithm of statistical parsimony using TCS vers. 1.21 [49]. The long-fragment indel of *Chs *sequences was treated as a one-mutation step and recoded. An analysis of molecular variance (AMOVA) was performed with Arlequin vers. 3.11 [50] in order to examine the components of genetic variation and the population structure.

Genetic differentiation between populations (pairwise *F*_ST_) was calculated by Arlequin [50] based on Wright's [51] equation. To examine the influence of geographic barriers on levels of gene flow, the isolation-by-distance (IBD) model was used. If the stepping-stone dispersal model was tenable for *C. kanehirae *(and then the reinforcement of the geographic distance was necessary when estimating using MIGRATE), the IBD pattern was assumed. The IBD model was examined by the Mantel test (*F*_ST _vs. geographic or altitudinal slope distance) to analyze the geographic effect on the genetic distribution. A reduced major axis (RMA) regression [52] was used to calculate the slope for the IBD test. Hellberg [52] indicated that the RAM regression is superior to the ordinary least squares regression for the IBD test based on simulation data. The IBD test considering both linear geographic distance and altitudinal slope was performed with the web-based computer program, IBDWS vers. 3.15 [53] (available at http://ibdws.sdsu.edu/~ibdws/).

### Intralocus recombination

In the following analyses, all sites within studied loci were assumed to share the same genealogical history. However, recombination is believed to be common in nuclear loci and to influence the outcome of genealogically based analyses [54]. Therefore, we assessed recombination events with the "four-gamete test" using DnaSP vers. 5.0 [48]. Although this test only evaluates the minimum number of intralocus recombination events, it is sensitive to the presence of recombinations. When the test of the dataset of each locus (*Chs *and *Lfy*) suggested an intralocus recombination, we discarded the sites within the recombination block and retained the longest possible contiguous unrecombined sequence for subsequent analyses.

### Migration rate through time

Because the incomplete migration-drift equilibrium was inferred based on the IBD results, the gene flow (*Nm*) estimated by the reciprocal of pairwise *F*_ST _by Wright's [51] equation under the assumption of the migration-drift equilibrium may have been incorrect. Hence, varying degrees of the migration rate through time had to be considered. We jointly estimated all immigration and emigration rates by the Metropolis-coupled Markov chain Monte Carlo (MCMCMC) strategy in the MIGRATE program vers. 3.0 [55]. Because the Mantel test revealed essentially no correlations between genetic and geographic distances (see "Results"), the assumption of an identical migration potential was adopted in the MIGRATE algorithm. The prior distributions of uniform *θ *and equal migration rates were incorporated into the Bayesian approach in the MIGRATE algorithm. *F*_ST _estimates were used as starting values for the initial analysis. For all other analyses, parameters at the end of the previous run were used as starting values for the next run until results converged at approximately the same values. We used settings of three long chains with 5 × 10^6 ^sampled and 5 × 10^3 ^recorded genealogies and discarded 25% of them at the beginning of a chain. Multiple long chains were combined for estimates. Adaptive heating was applied with temperature specifications of 1.0, 1.2, 1.5, and 3.0. Three replicates of the dataset were analyzed to calculate the average and confidence intervals. Because gene flow between populations estimated by pairwise *M*, which indicates the migration rate over the mutation rate, revealed confusing results (data not shown), four geographic regions based on the determination of Lin *et al*. [35] were used for the reanalysis.

### Inferring the historical demography

Signatures of population demographic changes (bottlenecks or expansions) in *C. kanehirae *were first examined by Tajima's *D *[56] and Fu's *F*s [57] statistics using DnaSP 5.0 [48] to see whether *C. kanehirae *data conformed to expectations of neutrality or departed from neutrality due to factors such as a population bottleneck or expansion. The examination of deviation from neutrality by both *D *and *F*s indices was based on 1000 coalescent simulations with consideration of the recombination rate using DnaSP. Expectations of these statistics are nearly zero in a constant-size population; significant negative values indicate a sudden expansion in population size, whereas significant positive values indicate processes such as a population subdivision or recent population bottlenecks. Second, demographic changes in *C. kanehirae *were also examined by calculating the raggedness index of the observed mismatch distribution for each of the populations according to the population expansion model implemented in Arlequin 3.1 [50]. This measure quantifies the smoothness of the observed mismatch distribution. Small raggedness values represent a population which has experienced sudden expansion whereas higher values of the raggedness index suggest stationary or bottlenecked populations [58,59]. Third, the demographic history of whether *C. kanehirae *underwent a range expansion was investigated by the spatial expansion model in Arlequin [50,60-62]. We employed parametric bootstrapping (1000 replicates) as implemented in Arlequin to test the goodness-of-fit of the observed mismatch distribution to that expected under the spatial expansion model using the sum of squared deviations (SSD) statistic for regional and pooled samples. A significant SSD value is taken as evidence of departure from the estimated demographic model of spatial expansion in subdivided populations [60,62] using Arlequin [50]. The model of spatial expansion assumes that subdivided populations expanded their distribution range and increased the total number of individuals [60,61]. It estimates tau (*τ*), the demographic expansion factor, with 95% confidence intervals, and the mutation parameter, *θ*, as well as the number of migrants (*M*). This least-squares estimation of *τ *is reliable but was found to be unreliable for estimating the migration parameter, *M *[63]. Therefore, we took the *M *estimate of > 1 together with a non-significant SSD value as evidence of spatial expansion. The time at which the expansion event took place was dated following the expression, *t *= *τ*/2 μk, where *τ *is the estimated number of generations since the expansion, μ is the mutation rate per site per generation, and k is the sequence length. A mutation rate of 1.5% per 10^6 ^years per site was used for both *Chs *[64] and *Lfy*. We assumed a mean generation time of 20 years for *C. kanehirae *in converting the time to expansion to years.

Patterns of historical demography can also be inferred from estimates of the effective population size over time using the BSP method [38] as implemented in BEAST vers. 1.4.8 [65] for estimating fluctuations in the effective population size. This method estimates a posterior distribution of effective population sizes through time via MCMC procedures, by moving backward until the time of the most recent common ancestor is reached. The constant population size coalescent model was the basic assumption used for this approach. A HKY model together with among-site rate heterogeneity across all branches but assuming a strict molecular clock was used for this calculation. Markov chains were run for 2.5 × 10^7 ^generations and were sampled every 1000 generations, with the first 2500 samples discarded as burn-in. Three replicates were run and combined to separately analyze the *Chs *and *Lfy *datasets. Other parameters were set as default values. TRACER vers. 1.4 [66] was used to visualize the posterior probabilities of the Markov chain statistics and to calculate a statistical summary of the genetic parameters.

## Results

### Genetic diversity

In total, 82 of 113 samples of *Chs *(72.5%) and 41 of 94 samples of *Lfy *(43.6%) were heterozygotes. Although all 226 alleles of *Chs *and 188 alleles of *Lfy *were unambiguously identified, 16 and 18 of them, respectively, were probably derived from *Taq *errors and were thus excluded from further analysis. PCR amplification resulted in 637 and 657 bp of the *Chs *gene sequence spanning exon 1 (1~18 bp), intron 1 (19~594 bp or a 20-bp indel), and exon 2 (595~637 bp), and 741 bp of the *Lfy *gene sequence spanning exon 2 (1~75 bp), intron 2 (76~692 bp), and exon 3 (693~741 bp). All *Chs *variations occurred within the region of the intron. In aligned *Lfy *sequences, two variable sites were found within exon 2, 29 variable sites were found within intron 2, and three variable sites were found within exon 3 (Additional file 1). The aligned lengths of 210 *Chs *sequences were 657 bp and 170 *Lfy *sequences were 741 bp, which yielded 36 and 35 haplotypes, respectively (Table 1, Additional file 1). Details of the genetic diversity of populations and regions are presented in Table 2. Among 34 polymorphic sites of *Chs*, 14 were substitutions and 20 were indels; whereas only one indel was found among 35 polymorphic sites of *Lfy*. Among all of the populations examined, nucleotide diversity (*π*) was 0.00716 and ranged from 0.00372 (population CP in the SE region) to 0.00865 (population ALS in the WC region) in *Chs *and was 0.00479 and ranged from 0 (population YF in the SE region) to 0.00838 (population FL in the SE region) in *Lfy*. Haplotype diversity (*Hd*) was 0.841 and ranged from 0.515 (population CP in the SE region) to 1.000 (population HNS in the SW region) in *Chs *and was 0.895 and ranged from 0.000 (population YF in the SE region) to 1.000 (population WC in the WC region) in *Lfy*. Haplotype and nucleotide diversities of the four geographic regions were also calculated: haplotype diversities (*Hd*) ranged from 0.679 (SE region) to 0.906 (WC region) in *Chs *and from 0.674 (NW region) to 0.896 (SE region) in *Lfy*; nucleotide diversities ranged from 0.00511 (SE region) to 0.00784 (SW region) in *Chs *and from 0.00260 (WC region) to 0.00625 (SE region) in *Lfy*. When comparing the genetic diversity of regions to each other, the haplotype diversity and nucleotide diversity of the SE region estimated for *Chs *were significantly lower than those of the other three regions, but the nucleotide diversity of the SE region estimated for *Lfy *was significantly higher than those of the other regions. In addition, the haplotype diversity of the NW region estimated for *Lfy *was significantly lower than that of the SE region (Table 2).

**Table 2:** Genetic diversity of 19 populations of *Cinnamomum kanehirae *estimated using *Chs *and *Lfy *sequences.

	*Chs*							
	
	*N*	*H*	*Hd*	*π *(× 10^-3^)	*θ *(*S*) (× 10^-3^)	Tajima's *D *(*P*)	Fu's *Fs *(*P*)	Raggedness index
Population								
KW	6	3	0.733 ± 0.155	6.91 ± 1.88	6.19 ± 3.42	-0.027(0.757)	0.094(0.944)	0.5067
NC	10	8	0.889 ± 0.075	7.50 ± 1.33	6.66 ± 3.20	-0.018(0.788)	-0.731(0.725)	0.0844
TH	6	4	0.800 ± 0.172	6.49 ± 2.84	7.56 ± 4.07	-0.112(0.274)	0.450(0.633)	0.6267
TP	10	5	0.844 ± 0.080	7.33 ± 0.93	5.55 ± 2.75	-0.022(0.974)	-0.665(0.886)	0.1368
ALS	10	6	0.844 ± 0.080	8.65 ± 1.08	6.66 ± 3.20	-0.296(0.976)	-0.551(0.937)	0.0756
CT	30	17	0.917 ± 0.030	7.20 ± 0.40	4.76 ± 1.95	-0.033(0.994)	-2.684(0.406)	0.0256
WC	5	4	0.800 ± 0.164	5.65 ± 1.45	4.52 ± 2.76	-0.041(0.987)	0.061(0.869)	0.2800
WH	5	4	0.900 ± 0.161	7.85 ± 2.20	8.29 ± 4.66	0.011(0.314)	-0.369(0.760)	0.3100
FKS	22	8	0.732 ± 0.090	8.09 ± 0.98	5.60 ± 2.35	-0.089 (0.969)	-0.486(0.803)	0.1135
HNS	5	6	1.000 ± 0.126	7.22 ± 2.51	8.29 ± 4.66	-0.043(0.149)	0.166(0.104)	0.1600
KS	21	7	0.786 ± 0.058	4.96 ± 0.33	3.05 ± 1.49	-0.053(0.993)	-1.000(0.903)	0.0986
TN	7	5	0.524 ± 0.209	3.89 ± 2.33	5.13 ± 2.80	-0.066(0.119)	0.357(0.843)	0.1950
CL	6	5	0.733 ± 0.155	5.23 ± 1.27	4.13 ± 2.44	0.022(0.970)	-0.082(0.933)	0.5067
CP	17	7	0.515 ± 0.145	3.72 ± 1.09	3.71 ± 1.79	-0.035(0.541)	0.105(0.509)	0.3279
FL	6	4	0.867 ± 0.129	7.64 ± 1.53	6.88 ± 3.75	0.010(0.833)	-0.451(0.878)	0.2756
HKL	16	5	0.650 ± 0.075	5.14 ± 0.46	3.31 ± 1.66	-0.062(0.989)	-0.281(0.961)	0.3197*
TML	10	4	0.778 ± 0.091	5.65 ± 0.85	4.44 ± 2.29	-0.041(0.923)	-0.335(0.931)	0.1590
YF	10	4	0.733 ± 0.101	5.44 ± 0.64	3.88 ± 2.06	-0.011(0.984)	-0.553(0.940)	0.2696
YL	8	3	0.714 ± 0.123	5.16 ± 0.82	3.63 ± 2.05	-0.023(0.995)	-0.115(0.958)	0.2041
Region								
NW	32	10	0.853 ± 0.039	7.35 ± 0.57	5.46 ± 2.15	-0.022(0.919)	-1.308(0.771)	0.0332
WC	50	20	0.906 ± 0.024	7.55 ± 0.31	4.56 ± 1.74	-0.048(0.994)	-3.671(0.583)	0.0114
SW	55	20	0.876 ± 0.026	7.84 ± 0.29	4.46 ± 1.69	-0.014(0.999)	-3.388(0.643)	0.0251
SE	73	14	0.679 ± 0.041	5.11 ± 0.21	3.23 ± 1.28	-0.034(0.953)	-1.019(0.676)	0.2150
Total	210	41	0.841 ± 0.019	7.16 ± 0.19	3.71 ± 1.25	-0.064(0.999)	-4.405(0.255)	0.0186

	***Lfy***							
	
	***N***	***H***	***Hd***	***π *(× 10^-3^)**	***θ *(*S*)** **(× 10^-3^)**	**Tajima's *D *(*P*)**	**Fu's *Fs *(*P*)**	**Raggedness index**

Population								
KW	6	4	0.867 ± 0.129	1.89 ± 0.47	1.78 ± 1.23	-0.022(0.682)	-0.282(0.197)	0.1867
NC	12	3	0.318 ± 0.164	1.04 ± 0.54	1.34 ± 0.88	-0.051(0.262)	0.269(0.480)	0.4860
TH	8	3	0.714 ± 0.123	2.12 ± 0.37	1.56 ± 1.06	0.027(0.971)	-0.800(0.949)	0.1225
TP	7	4	0.857 ± 0.102	6.56 ± 1.61	6.62 ± 3.41	-0.063(0.519)	-0.356(0.915)	0.1474
ALS	4	2	0.667 ± 0.204	0.90 ± 0.28	0.74 ± 0.74	-0.002(0.883)	0.065(0.852)	0.5556
CT	3	2	0.667 ± 0.314	0.90 ± 0.42	0.90 ± 0.90	-	-	0.5556
WC	4	4	1.000 ± 0.177	3.15 ± 0.82	2.95 ± 2.01	-0.044(0.806)	0.059(0.067)	0.2222
WH	6	2	0.333 ± 0.215	0.45 ± 0.29	0.59 ± 0.59	-0.003(0.240)	0.154(0.568)	0.2222
FKS	19	4	0.456 ± 0.132	1.50 ± 0.46	1.93 ± 1.04	-0.035(0.277)	0.150(0.550)	0.3502
HNS	8	2	0.571 ± 0.094	2.32 ± 0.38	1.56 ± 1.06	-0.007(0.994)	-0.171(0.985)	0.8367*
KS	20	11	0.916 ± 0.038	4.11 ± 0.93	5.33 ± 2.24	-0.042(0.188)	-0.797(0.045)	0.0680
TN	3	2	0.667 ± 0.314	6.31 ± 2.97	6.31 ± 4.23	-	-	1.0000
CL	6	3	0.733 ± 0.155	6.49 ± 1.30	5.33 ± 2.95	-0.015(0.957)	-0.091(0.973)	0.3467
CP	13	8	0.885 ± 0.070	7.87 ± 1.06	8.27 ± 3.56	-0.035(0.406)	-1.884(0.812)	0.1142
FL	5	4	0.900 ± 0.161	8.38 ± 1.93	7.78 ± 1.33	-0.003(0.822)	-0.207(0.792)	0.3300
HKL	20	7	0.789 ± 0.068	5.69 ± 0.59	5.33 ± 2.24	-0.037(0.645)	-2.025(0.945)	0.5288**
TML	8	4	0.750 ± 0.139	2.22 ± 0.36	1.56 ± 1.06	-0.029(0.993)	-1.148(0.724)	0.1263
YF	8	1	0.000 ± 0.000	0.00 ± 0.00	0.00 ± 0.00	-	-	0.0000
YL	10	3	0.511 ± 0.164	1.98 ± 1.13	2.87 ± 1.57	-0.035(0.113)	0.263(0.796)	0.1669
Region								
NW	33	6	0.674 ± 0.078	2.81 ± 0.66	4.33 ± 1.73	-0.094(0.130)	-0.088(0.683)	0.0435
WC	17	9	0.890 ± 0.054	2.60 ± 0.33	2.40 ± 1.24	0.000(0.659)	-6.344(0.634)	0.0614
SW	50	15	0.871 ± 0.032	3.11 ± 0.49	4.83 ± 1.76	-0.064(0.106)	-1.005(0.012)	0.0448
SE	70	21	0.896 ± 0.019	6.25 ± 0.36	7.29 ± 2.32	0.000(0.254)	-6.017(0.606)	0.0553
Total	170	35	0.895 ± 0.015	4.79 ± 0.31	8.05 ± 2.22	-0.041(0.062)	-3.496(0.001)	0.0211

Populations and regions are defined in Table 1.

Tajima's *D *and Fu's *F*s were estimated based on the coalescent simulations with 1000 replicates in consideration of recombination.

* *P *< 0.05; ** *P *< 0.00001 for the raggedness index reject the null hypothesis of expectation under a sudden demographic expansion model.

*N *is the number of sequences; *H *is the number of haplotypes; *Hd *is the haplotype diversity; *π *and *θ *are the nucleotide diversity estimates.

### Population structure and gene flow

**Figure 2:**
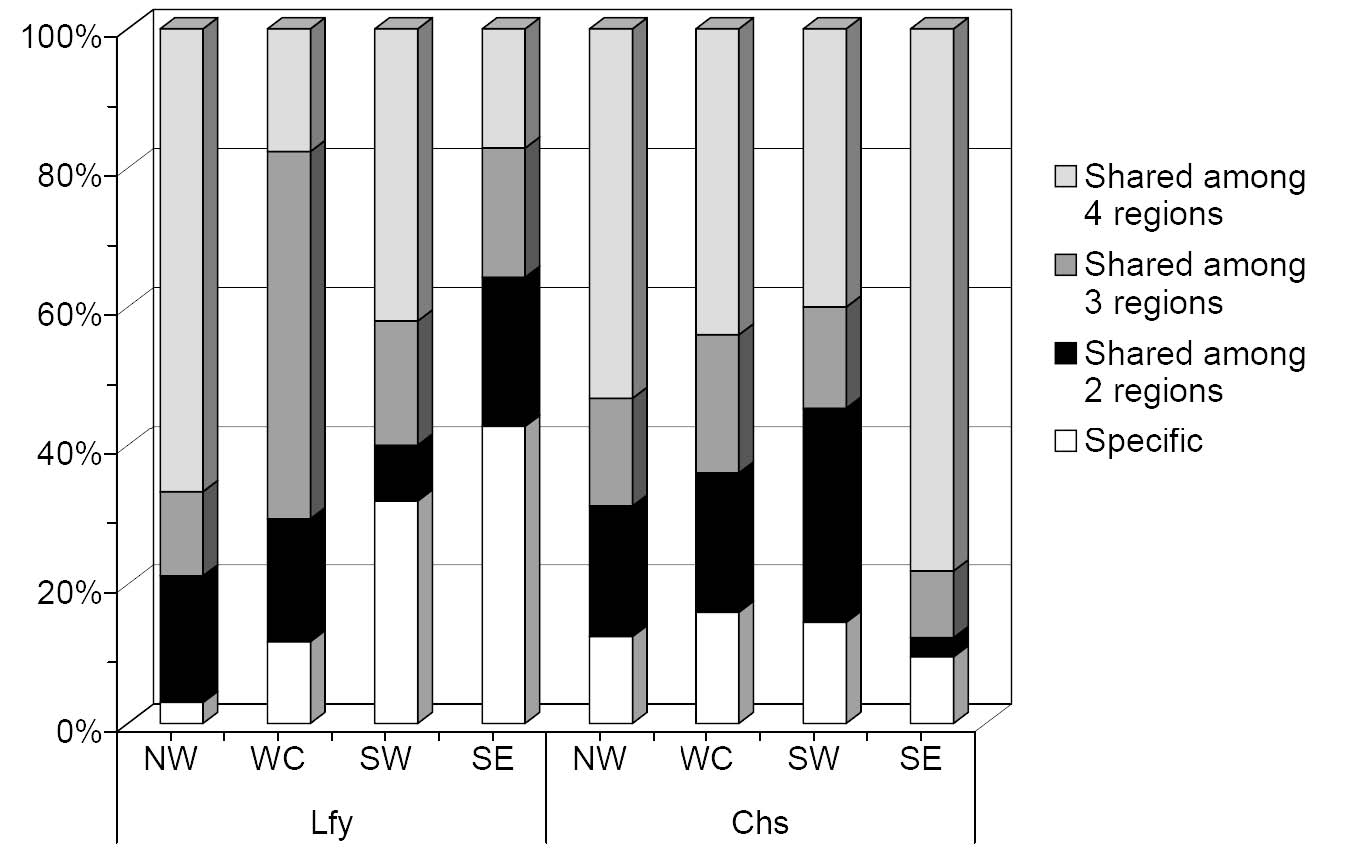
**Proportions of common and rare haplotypes of *Cinnamomum kanehirae *among the four geographic regions**. The white bar indicates the proportion of haplotypes that specifically appeared in only one of the four regions; the dark bar is the proportion of haplotypes that also appeared in one of the three other regions; the intermediate-gray bar is the proportion of haplotypes that also appeared in two of the three other regions; and the light-gray bar indicates the proportion of haplotypes that appeared in all four regions.

Mean population pairwise *F*_ST _values were 0.101 and 0.293 for the *Chs *and *Lfy *sequences, respectively (Additional file 2). The haplotype network depicted no clear patterns of population or regional genetic structuring (Additional file 3). Mean *F*_ST _values for regional comparisons were 0.033 and 0.086 for the *Chs *and *Lfy *sequences, respectively (Table 3). Although the pairwise *F*_ST _estimated from two nuclear DNA sequences showed discrepancies on the levels of genetic differentiation among regional comparisons, both *Chs *and *Lfy *pairwise *F*_ST _matrices revealed higher genetic differentiation when SE was compared to the regions west of the CMR, but lower levels of genetic differentiations were found when only regions west of the CMR were compared (Table 3). The conspicuous level of genetic differentiation between regions on either side of the CMR indicates its barrier effect. A lack of migration-drift equilibrium can be assumed according to the result of the IBD tests (see below). Gene flow (*Nm*) estimated using Wright's model [51] which assumes migration-drift equilibrium was not appropriate for estimating magnitudes of gene flow. Therefore, gene flow varying over time was assumed when estimating the level of gene flow due to differential levels of common haplotype sharing among regions west of the CMR and across the CMR (Figure 2). We applied the Bayesian approach implemented in MIGRATE [55] to estimate the magnitudes and directions of gene flow between pairs of regional samples. The high number of migrants (*M*) calculated by the Bayesian approach indicated frequent gene flow, which was not hindered by geological interruption (Figure 1). The Bayesian analysis of migration showed that the SE region may have served as a dispersal source southwards in recent times (Figure 1).

**Table 3:** Pairwise *F*_ST _estimates among the four geographic regions of *Cinnamomum kanehirae*.

*F*_ST_	NW	WC	SW	SE
NW	-	0.018	0.006	0.175***
WC	0.006	-	0.032	0.187**
SW	0.016	0.012	-	0.186***
**SE**	0.047*	0.042*	0.073*	-

The bold values indicate comparisons between the SE and other regions.

Above the diagonal: estimates for *Lfy*.

Below the diagonal: estimates for *Chs*.

Geographic regions are defined in Figure 1.

Significant *F*_ST _*P *values: * *P *< 0.05; ** *P *< 0.01; *** *P *< 0.00001.

**Figure 3:**
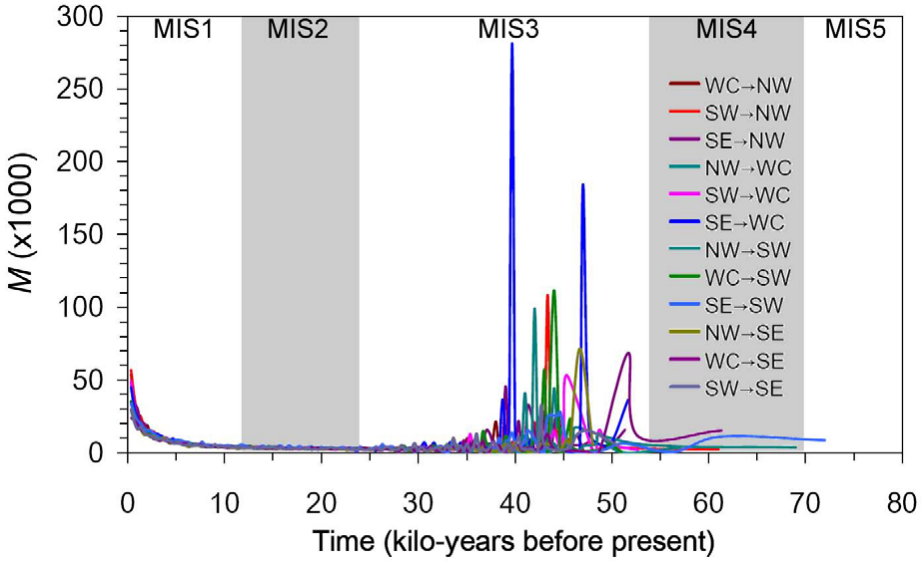
**The number of migrants through time**. Amplitudes of migrations through time are indicated by the parameter *M *multiplied by one thousand.

At the entire population and regional scales, lack of correlations between genetic (*F*_ST_) and geographic distances (both linear and altitudinal slope) was a common phenomenon according to the Mantel test, and only the SE-SW comparison showed a marginally significant correlation between genetic distance and linear geographic distance (*r *= 0.314, *P *= 0.049 for *Chs *sequences and *r *= 0.295, *P *= 0.047 for *Lfy *sequences, 1000 permutations, one-tailed test). If we consider the altitudinal slope, then the NW-SE and NW-WC comparisons showed significant correlations between genetic and geographic distances for *Chs *(*r *= 0.341, *P *= 0.011; *r *= 0.427, *P *= 0.044, respectively; 1000 permutations, one-tailed test), whereas no significant correlation was found based on *Lfy *sequences. In addition, we also estimated the most probable time at which gene flow occurred by the Bayesian approach performed using MIGRATE [55], and high probabilities of gene flow were found for each comparison during the last interglacial (Figure 3). This result indicates a trend of gene flow that occurred in the recent past or else a large, stable population size of this species in the past.

The AMOVA among the four geographic regions and between the east-west regions had similar results of a high proportion of the overall genetic variation being attributed to within-population levels (Table 4). On average, a relatively low percentage of the total genetic variation was attributable to among-region comparisons for both the four geographic regions and east-west regions. Nevertheless, the level of among-region genetic differentiation was significant when comparing eastern to western populations (Φ_CT _= 0.044, *P *< 0.01 for *Chs *and Φ_CT _= 0.170, *P *= 0.01 for *Lfy*). Significant genetic differentiation was also found in both sets of comparisons at the hierarchical level among populations within regions (Φ_SC _= 0.050 and Φ_SC _= 0.042 for *Chs *and Φ_SC _= 0.230 and Φ_SC _= 0.207 for *Lfy*, respectively, for comparisons of the four geographic regions and the eastern-western regions with *P *< 0.05). Moreover, genetic differentiation among populations was also significant for both sets of comparisons (Φ_ST _= 0.067 and Φ_ST _= 0.084 for *Chs*, and Φ_ST _= 0.299 and Φ_ST _= 0.341 for *Lfy*, respectively for comparisons of the four geographic regions and eastern-western regions with *P *< 0.01). When comparing the three geographic regions west of the CMR, both *Chs *and *Lfy *revealed no genetic structuring among regions, but a certain level of significant genetic structuring was found at the hierarchical levels among populations within regions and among populations (Φ_SC _= 0.080 and Φ_ST _= 0.053 for *Chs*, and Φ_SC _= 0.163 and Φ_ST _= 0.130 for *Lfy*, respectively). Although most comparisons of genetic structure were significant, the relatively shallow genetic structure on average especially when populations west of the CMR were compared represents a scenario of unhindered gene flow incompatible with the rugged geographic topology. The relatively shallow population structure was also supported by the mixed clustering relationships of populations (data not shown) and again reflects the large, contiguous distribution of this species in the past.

**Table 4:** Summary of the analysis of molecular variance (AMOVA).

Source of variation	df	**S.S**.	Variance component	% variation	Fixation indices	*P *value
(1) 4 regions						
*Chs*						
Among regions	3	39.775	0.091	1.76	Φ_CT _*= *0.01757	0.10362
Among populations within regions	15	110.731	0.255	4.92	Φ_SC _*= *0.05010	0.03715*
Within populations	191	922.56	4.83	93.32	Φ_ST _*= *0.06679	0.008*
Total	209	1073.07	5.176	100		
*Lfy*						
Among regions	3	38.162	0.181	9.07	Φ_CT _*= *0.09071	0.06843
Among populations within regions	15	74.078	0.416	20.89	Φ_SC _*= *0.22976	< 0.00001*
Within populations	151	210.737	1.396	70.04	Φ_ST _*= *0.29963	< 0.00001*
Total	169	322.976	1.993	100		
(2) E and W						
*Chs*						
Among regions	1	29.693	0.23	4.36	Φ_CT _*= *0.04364	0.00978*
Among populations within regions	17	120.813	0.214	4.05	Φ_SC _*= *0.04239	0.04203*
Within populations	191	922.56	4.83	91.58	Φ_ST _*= *0.08418	0.00489*
Total	209	1073.067	5.274	100		
*Lfy*						
Among regions	1	35.415	0.35925	16.95	Φ_CT _*= *0.16951	0.01075*
Among populations within regions	17	76.824	0.36443	17.2	Φ_SC _*= *0.20706	< 0.00001*
Within populations	151	210.737	1.39561	65.85	Φ_ST _*= *0.34147	< 0.00001*
Total	169	322.976	2.11929			
(3) Three western regions						
*Chs*						
Among regions	2	10.082	-0.16840	-2.98	Φ_CT _= -0.02983	0.95205
Among populations within regions	9	90.017	0.46911	8.31	Φ_SC _= 0.08068	0.02513*
Within populations	125	668.178	5.34543	94.67	Φ_ST _= 0.05326	0.02811*
Total	136	768.277	5.64613			
*Lfy*						
Among regions	2	2.747	-0.04460	-3.91	Φ_CT _= -0.3910	0.67097
Among populations within regions	9	22.460	0.19358	16.97	Φ_SC _= 0.16330	< 0.00001*
Within populations	88	87.283	0.99186	86.94	Φ_ST _= 0.13058	< 0.00001*
Total	99	112.490	1.14083			

* Statistically significant (*P *< 0.05)

Three comparisons for the AMOVA were made for both *Chs *and *Lfy *loci: (1) four geographic regions as defined by Lin et al. [35], (2) the eastern (E) and western (W) parts separated by the Central Mountain Range (CMR), and (3) the three geographic regions west of the CMR

### Demographic history

Neutrality tests of Tajima's *D *revealed non-significant negative values in many individual populations, pooled samples and the four regional samples for both the *Chs *and *Lfy *datasets (Table 2). Non-significant negative Fu's *F*s values were also obtained for all populations, regional samples, and the pooled samples for *Chs *data. Similar results were also seen for most populations and regional samples with *Lfy *data; however, significant negative Fu's *F*s values (*P *< 0.05) in the KS population, SW regions, and pooled samples were found (Table 2). Fu's *F*s is considered to be more sensitive in detecting population expansion and suggesting possible past population growth, but the expansion may have been restricted to separate local areas that resulted in the non-significant negative Fu's *F*s values for most populations and regions. Moreover, either population reduction, population subdivision, a recent bottleneck, or migration which resulted in secondary contact among previously differentiated lineages could have caused the discrepancy in detecting population expansion using the pooled samples with these two neutrality test statistics [67,68]. Non-significant or small negative values of Tajima's *D *and Fu's *F*s were found in many individual populations suggesting stationary in *C. kanehirae *populations. We also calculated the raggedness index under the demographic expansion model for each population and found that many individual populations had a high raggedness index (Table 2). We further analyzed the mismatch distribution under the spatial expansion model considering population subdivision [60-62]. Analysis of the mismatch distribution showed a multimodal pattern characteristic of population differentiation which corresponds to demographic growth and a subsequent bottleneck [[61], data not shown]. It is probable that loss of haplotypes due to the recent bottleneck would have caused the multimodal mismatch distribution. According to the mismatch distribution analysis, the hypothesis of spatial expansion could not be rejected in pooled samples (SSD = 0.021, *P *= 0.502 for *Chs *and SSD = 0.011, *P *= 0.38 for *Lfy*, Table 5). A good fit of the spatial expansion was also observed for regional samples according to the non-significant SSD values; however, different expansion times of regional samples estimated by *τ *were found due to different coalescent histories of *Chs *and *Lfy *sequences (Table 5). The relatively longer expansion time and smaller migration rate of the NW region for both sequence sets (*τ *= 8.95 and *M *= 0.02 for *Lfy*; *τ *= 25.8 and *M *= 0.89 for *Chs*) implies a far-distant spatial expansion for older lineages of NW regional samples. In contrast to NW samples, SE regional samples revealed a relatively shorter expansion time and larger migration rate (*τ *= 2.50 and *M *= 8.08 for *Lfy*; *τ *= 5.5 and *M *= 1.65 for *Chs*), which indicates a recent spatial expansion.

**Table 5:** Estimated spatial expansion parameters for *Cinnamomum kanehirae*.

Estimated parameters/Geographic region	NW	WC	SW	SE	Western	Total
*Chs*						
Tau (95% CI)	25.8 (0.37~54.22)	22.4 (0.75~131.46)	22.1 (0.47~149.32)	5.5 (1.45~10.04)	22.6 (0.62~143.08)	5 (1.22~14.06)
Theta (95% CI)	5.19 (0.00072~10.97)	9.00 (0.19~13.69)	9.74 (0.19~14.97)	0.49(0.00072~2.12)	8.73(0.09066~13.99)	2.785 (0.00072~5.71)
Migration rate (95% CI)	0.89 (0.232~99999)	0.52 (0.384~99999)	0.43 (0.313~99999)	1.65 (0.343~9.10)	0.52 (0.315~81.79)	2.27 (0.572~16.20)
SSD	0.0232	0.01866	0.02165	0.06415	0.0137	0.02117
*P*-value	0.505	0.566	0.406	0.268	0.649	0.502
*Lfy*						
Tau (95% CI)	8.95 (0.38-9.88)	2.10 (0.89-2.93)	2.00 (1.11-2.44)	2.50 (0.99-11.77)	1.40 (0.99-2.65)	1.00 (0.52-10.21)
Theta (95% CI)	2.09 (0.00-2.66)	0.00 (0.00-1.20)	0.00 (0.00-0.88)	4.19 (0.00-8.43)	0.44 (0.00-0.98)	3.11 (0.00-5.93)
Migration rate (95% CI)	0.02 (0.48-99999)	99999 (7.08-99999)	99999 (12.67-99999)	8.08 (1.14-168.21)	99999 (12.79-99999)	74.76 (1.08-99999)
SSD	0.013	0.004	0.003	0.032	0.003	0.011
*P*-value	0.60	0.55	0.24	0.07	0.15	0.38

Confidence intervals (CI), α = 0.05.

SSD: sum of squared deviations.

Geographic regions are defined in Figure 1.

Values were obtained from the mismatch distribution under the spatial expansion model.

**Figure 4:**
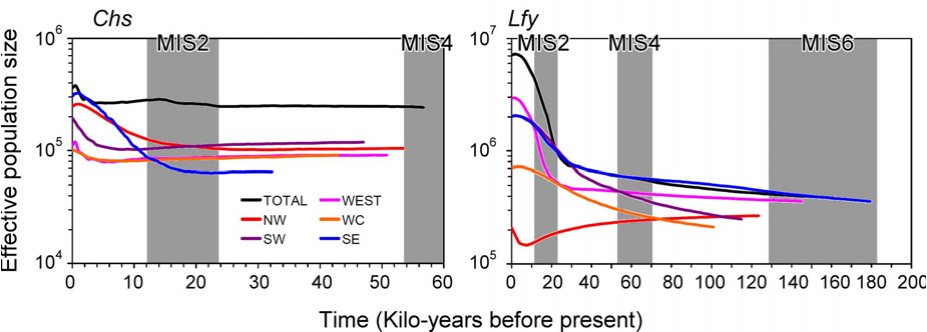
**Bayesian skyline plot revealing the demographic trends of *Chs *and *Lfy *intron lineages of *Cinnamomum kanehirae***. The population size was measured as the effective population size per generation. MIS, marine isotope stage.

The BSP analysis suggested a past increase in the effective population size of *C. kanehirae *for both nuclear markers (Figure 4, Additional file 4). An increase in the effective population size inferred by *Chs *was smaller than that inferred by *Lfy*. Moreover, the time of population growth inferred by *Chs *was later than that inferred by *Lfy*. Slight differences in trends of the population size increase and expansion time are likely due to different coalescent histories of the two loci and uncertainty of molecular dating [69]. On the other hand, a clear recent bottleneck was revealed by the pooled samples of *Chs *sequences, but *Lfy *data showed only a slight decrease in population size after the last glacial maximum (LGM) expansion. In addition, regional samples of western, WC, and SE revealed a more-obvious population decline for *Chs *than for *Lfy *(Figure 4). Moreover, NW samples displayed a history of a prolonged population decline followed by a recent sudden expansion based on *Lfy *variations.

## Discussion

### Genetic diversity

Historical demographic events and population sizes of most natural populations fluctuated and left traces in patterns of their genetic diversity. In this study, *C. kanehirae *had higher or comparable nucleotide diversities for both *Chs *and *Lfy *or either one of them separately compared to the nucleotide diversity in population studies of nuclear genes such as *Chs *in the *Hippophaë rhamnoides *species complex [70], the glyceraldehyde-3-phosphate dehydrogenase (*G3pdh*) gene in *Q. glauca *[71] and the nucleotide diversity of *G3pdh *in *Manihot esculenta *[72], the *PHYP *and *PHYO *loci in *Pinus sylvestris *[73], and the fruit vacuolar invertase gene in *Solanum pimpinellifolium *[74]. Although the comparison of nucleotide diversity using nuclear DNA sequence data might not be complete, the high level of allozyme variation found also suggests a great potential for high genetic variation in nuclear DNA sequences of *C. kanehirae *[35]. High nucleotide diversity of nuclear genes may reflect a long evolutionary history of a large, stable population, or mixing of differential lineages via range expansion [75-77] and is consistent with field surveys of the contiguous range distribution of *C. kanehirae *in the past [25,26].

### Historical and recent demography of *Cinnamomum kanehirae*

Overall, it can be seen that the demographic history of *C. kanehirae *is far more complex than previously thought, with evidence of historical range expansion and a very recent bottleneck (Table 2, Figure 4). While population expansion was rejected by Tajima's *D *and Fu's *F*s test statistics in most population and regional samples, the high raggedness index estimated for individual populations suggests the occurrence of bottleneck events [78]. Population bottlenecks were also evident when analyzed with the BSP (Figure 4). On the other hand, the mismatch analyses failed to reject a spatial expansion model for all datasets including pooled and regional samples (Table 5). Historical spatial expansion was also revealed by the BSP analysis (Figure 4). From these results the demographic history of *C. kanehirae *conformed to the hypothesis of past spatial range expansion followed by a very recent population bottleneck. However, the past spatial range expansion might not display a molecular signature of a unimodal distribution characteristic of sudden expansions in un-subdivided populations, even though populations had expanded by several orders of magnitude after the past expansion [61] and was evident through analysis with the MIGRATE software, which might reflect a historically large stable population of *C. kanehirae *(Figure 3). The level of nucleotide diversity of *C. kanehirae *might therefore be a species-associated feature, and also a result of its past evolutionary history as well as contemporary events.

The climate during interglacial periods in Taiwan was mostly humid and warm (in contrast to glacial periods) [79,80], and therefore habitats at different elevations were appropriate for the growth of *C. kanehirae*. Population expansion after the LGM was inferred for several plant species including *C. kanehirae *based on cpDNA variations [21,37,40,81]. Further information on the demographic history of *C. kanehirae *was revealed by nuclear DNA variations. It is likely that larger genetic variability that accumulated in nuclear markers coalesced into a longer genealogy relative to cpDNA, reflecting greater sensitivity in revealing insights into both recent and older events. Moreover, *C. kanehirae *is an insect-pollinated hermaphrodite with male and female flowers at different times possibly promoting outcrossing and therefore at least a 2-fold higher effective population size of nuclear loci relative to cpDNA makes it more sensitive to population oscillations. The vegetational history traced by pollen records illustrates rapid growth of *Alnus*, *Liquidambar*, *Mallotus*, *Trema*, and the Chenopodiaceae in subtropical forests of Taiwan [82]. Characteristics of a slow growth rate and long generation time (approximate 20 years) may be limiting factors in habitat establishment for *C. kanehirae *when competing with other plant species, which thus reinforced the present-day bottleneck.

Loss of genetic diversity due to anthropogenic factors might not be apparent because of the high genetic diversity that accumulated during past range expansions and resulted in only a slight decline in the recent effective population size as estimated by the BSP analysis. Nevertheless, this small decline in the recent population size reveals a commencing phase of a bottleneck in *C. kanehirae *inflicted by anthropogenic factors. The past increase in gene flow and spatial expansion should have assisted the dispersal of population- and region-specific haplotypes; however, human interventions including over-deforestation and habitat destruction caused declining of population size and fewer opportunities for colonization of these specific haplotypes.

### Contemporary genetic structure and gene flow

*Cinnamomum kanehirae *is a plant species with bird-dispersed seeds. Forest birds in the warm period of the last interglacial [83] could have facilitated seed dispersal of Lauraceae species and accelerated gene flow between *C. kanehirae *populations. *Cinnamomum kanehirae *has a similar pattern of diversity distribution as that of *Machilus thunbergii *(Lauraceae) based on cpDNA variations with most populations possessing the ancestral haplotype [40,84]. A shared ancestral polymorphism was the cause of the lack of genetic differentiation among populations in these species. In this study, relatively low levels of genetic differentiation (*F*_ST_) and a high degree of gene flow were also found by estimates of *M *based on nuclear DNA variations. Moreover, the haplotype network depicted no clear patterns of geographic structuring, further supporting the regional migrations of haplotypes (Additional file 3). The overall spatial genetic differentiation estimated among all *C. kanehirae *populations was not purely stochastic, but instead probably reflects the effect of secondary contacts through migration due to range expansion after the LGM (Figure 3, Table 5).

Rates and patterns of dispersal and migration between populations will affect the genetic structure of species: the higher the dispersal ability, the lower the population structuring [85]. The AMOVA revealed a certain level of significant genetic structuring in *C. kanehirae *populations, but the level of the genetic structuring of the two nuclear DNA loci assayed was relatively low on average at all hierarchical levels. Relatively low levels of genetic differentiation in *C. kanehirae*, especially among populations west of the CMR (Table 3), can be attributed to two possibilities: first, gene exchange between long-term isolated populations and a second, recent fragmentation of a large population with recurrent gene flow. A similar consequence of a lack of genetic structuring and low population differentiation was observed for these two possibilities; however, there were slight differences in the outcomes. In the situation of long-term isolation, each separate population would have independently experienced genetic drift, and resulted in high frequencies of rare haplotypes in each population [86]. On the other hand, if the populations were recently separated, there would insufficient time for genetic drift to have occurred in each small fragmented population, and most populations would share common haplotypes [87]. Sharing of haplotypes was frequently found among populations and among regions (Table 1, Figure 2), although some haplotypes were region- and/or population-specific, however, with relatively low proportions. Therefore, the hypothesis of recent fragmentation of a large population with recurrent gene flow is highly probable. A general lack of IBD, at a regional scale to the west of the CMR, is typically considered to be the result of a large, stable population with historically recurring gene flow, which was further supported by the high migration rates among both populations and regions (Figure 3).

The implications of incomplete drift-migration equilibrium can further explain the contemporary genetic structure of *C. kanehirae*, which is the outcome of the interaction between inherent past spatial expansion with the barrier effect of the CMR and recent bottleneck events. Taiwan is a continental island which began to rise approximately 4 Mya by tectonic compression of the Eurasian and Philippine Sea Plates. The CMR geographically separates the landscape into eastern and western parts [13]. The barrier effect of gene flow by the CMR in Taiwan was observed in several plant and animal studies [37,81,88]. The AMOVA indicated some level of geographic structuring resulted in haplotypes specific to either the eastern or western populations of *C. kanehirae*, which might be related to the barrier effect of the CMR, and this was statistically supported (Table 4). The occurrence of population- or region-specific haplotypes may have resulted from a recent bottleneck event due to human interventions that caused the loss of haplotypes in other populations and regions; however, the possible failure to discover haplotypes due to the small sample size cannot be excluded. Historically, recurrent gene flow between populations, across geographic regions and across the CMR possibly diminished the level of genetic differentiation and the influence of the CMR barrier. Therefore, common haplotype distributions between geographic regions and relatively shallow genetic structuring are the result of historical gene flows. Under the drift-migration equilibrium, patterns of exclusivity are not expected, particularly for a large population size or a short isolation time. In this scenario, common haplotypes should be more abundant than private ones in an individual population. However, rare or population-specific haplotypes occurred in almost all of the populations examined (Table 1).

### Dispersal after the LGM

Historical and extrinsic ecological events can leave signatures in the spatial genetic structure of a species and provide insights into understanding the species' evolutionary history [75]. In Taiwan, six subtropical tree species have been phylogeographically studied using cpDNA variations, and the SE part of Taiwan was inferred to be a major diversity center based on average *F*_ST _divergences from populations of other regions [22,37,40,71,81,84,89], and that was further suggested by this study because a higher average *F*_ST _divergence from other regions was found when the SE was compared to other regions (Table 3). However, the SE region being postulated as a diversity center in this way may only reflect its long isolation from other regions, which was suggested by the high level of regional-specific haplotypes and very recent expansion southward. In contrast, the southerly expansion event of the NW region to the west of the CMR was older according to the TMRCA estimates (Table 5). The trend of a temperature rise after cold climatic periods of the Pleistocene was inferred to have occurred from south to north assisted by the warm Kuroshio Current that flow northward offshore of the east coast of Taiwan [90]. Although the South China Sea Warm Current flowed through the Taiwan Strait after the LGM when sea levels again rose, the magnitude of the temperature rise might not have been significant, as the area west of the CMR faces cold fronts from the north as well as the barrier effect of the CMR in receiving warm temperatures from the south carried by the Kuroshio Current. Thus, a deferred timing of the temperature rise may have occurred in western populations of *C. kanehirae *(except populations in the SW region). The onset of a temperature rise after the LGM for populations located in the SE region preceded that of populations located in other regions, thereby facilitating the migration from SE to SW regions by *C. kanehirae*.

## Conclusions

Distributions of genetic variations in wild populations provide insights into a species' evolutionary history and its future continuity [75,91]. *Cinnamomum kanehirae *is confronted with conditions of being rare, fragmented, and threatened at the present time, consistent with the genetic survey that revealed a pattern of population growth followed by an ongoing bottleneck. Despite the declining effective population size of *C. kanehirae*, the genetic diversity of remnant individuals showed no dramatic reduction in genetic diversity. In *C. kanehirae*, a tree with a long generation time, the rapid population decline probably began decades ago due to anthropogenic interventions for two or more generations. Thus information on the loss of genetic diversity might not be spontaneously revealed, but will probably be seen within two or more generations if the populations are not restored. In light of the declining effective population size, the condition of the true population size of *C. kanehirae *at present may be far more severely threatened. In addition to the continuing illegal selective cutting for cultivating the medicinal fungus, *A. cinnamomea*, the loss of habitat is another serious issue contributing to population declines of *C. kanehirae*. Elevations where *C. kanehirae *grows range 200~2000 m, and overdevelopment for farming vegetables, tea, and fruit crops has destroyed the natural habitats of primeval forests, including habitats of *C. kanehirae*. Therefore, loss of the population size of *C. kanehirae *was mainly caused by human overexploitation. In consequence, it is essential that this valuable species be conserved through habitat restoration, which can advance the restoration of the effective population size of *C. kanehirae*. Further field surveys to locate plants and grafts for *ex situ *conservation are another requirement to conserve this species. Management strategies should also consider reinforcing local populations with individuals originating from populations showing the closest genetic relatedness to produce larger population with higher genetic variability. In summary, conservation strategies must therefore aim for habitat restoration and remnant diversity preservation, and avoid the occurrence of genetic inbreeding in populations of *C. kanehirae*.

## Authors' contributions

SYH and TPL conceived the research; DCK, CCL, and KCH collected the data; PCL analyzed the data and wrote the first draft; SYH led the writing. All authors read and approved the final manuscript.

## Supplementary Material

Additional file 1 **Polymorphic sites among haplotypes at *Chs *and *Lfy***. The table provides polymorphic information for both the *Chs *and *Lfy *loci for each haplotype. Click here for file

Additional file 2 **Pairwise F_ST _of 19 Cinnamomum kanehirae populations**. The table provides the pairwise genetic distance matrix obtained using *Chs *and *Lfy *DNA. Click here for file

Additional file 3 **Phylogenetic network constructed by both *Chs *and *Lfy *sequences**. The figure shows the haplotype networks constructed using a statistical parsimony approach. The long-fragment indel of *Chs *sequences was treated as one-mutation step and recoded. The limit of 90% connection was used to estimate the network. Four geographic regions were indicated proportionally with four colors. Click here for file

Additional file 4 **Changes in effective population size in regional and pooled samples of Cinnamomum kanehirae**. The figure shows the effective population size per generation for regional and pooled samples of Cinnamomum kanehirae based on Chs and Lfy datasets. The solid line represents mean values of the estimated effective population size with lower (0.05) and upper (0.95) bounds. Click here for file
