# Supplementary material for: Historical spatial range expansion and a very recent bottleneck of Cinnamomum kanehirae Hay. (Lauraceae) in Taiwan inferred from nuclear genes
Source: BMC Evol Biol. 2010 Apr 30;10:124. doi: 10.1186/1471-2148-10-124 (PMC2880300; doi:10.1186/1471-2148-10-124)
Supplement: Additional file 1 — Polymorphic sites among haplotypes at Chs and Lfy. The table provides polymorphic information for both the Chs and Lfy loci for each haplotype. [file 1471-2148-10-124-S1.DOC]

**Table S1:** ***Polymorphic sites among haplotypes at* Chs *and* Lfy***.*

| *Chs* |  | 1 | 1 | 1 | 2 | 2 | 2 | 2 | 2 | 3 | 3 | 3 | 4 | 4 | 5 | 5 | 5 | 5 | 5 | 5 | 5 | 5 | 5 | 5 | 5 | 5 | 5 | 5 | 5 | 5 | 5 | 5 | 5 | 5 |  |
| --- | --- | --- | --- | --- | --- | --- | --- | --- | --- | --- | --- | --- | --- | --- | --- | --- | --- | --- | --- | --- | --- | --- | --- | --- | --- | --- | --- | --- | --- | --- | --- | --- | --- | --- | --- |
|  | 4 | 0 | 3 | 5 | 0 | 0 | 3 | 5 | 8 | 3 | 3 | 3 | 1 | 6 | 3 | 3 | 3 | 3 | 3 | 4 | 4 | 4 | 4 | 4 | 4 | 4 | 4 | 4 | 4 | 5 | 5 | 5 | 5 | 5 |  |
|  | 0 | 9 | 5 | 3 | 1 | 6 | 9 | 9 | 5 | 4 | 5 | 6 | 0 | 0 | 5 | 6 | 7 | 8 | 9 | 0 | 1 | 2 | 3 | 4 | 5 | 6 | 7 | 8 | 9 | 0 | 1 | 2 | 3 | 4 |  |
| Hap_1 | C | G | G | T | A | G | C | A | A | T | A | A | T | T | - | - | - | - | - | - | - | - | - | - | - | - | - | - | - | - | - | - | - | - |  |
| Hap_2 | . | . | A | A | G | A | T | G | T | A | . | . | A | C | - | - | - | - | - | - | - | - | - | - | - | - | - | - | - | - | - | - | - | - |  |
| Hap_3 | A | C | . | . | G | . | . | . | T | A | . | . | A | C | - | - | - | - | - | - | - | - | - | - | - | - | - | - | - | - | - | - | - | - |  |
| Hap_4 | . | . | . | A | G | A | T | G | T | A | . | . | A | C | - | - | - | - | - | - | - | - | - | - | - | - | - | - | - | - | - | - | - | - |  |
| Hap_5 | . | . | . | . | G | . | . | . | . | . | . | . | A | . | T | A | T | G | A | A | T | T | G | G | T | C | A | A | A | A | T | T | G | A |  |
| Hap_6 | . | . | . | . | . | . | . | . | . | . | . | . | . | . | T | A | T | G | A | A | T | T | G | G | T | C | A | A | A | A | T | T | G | A |  |
| Hap_7 | . | . | . | . | G | . | . | . | T | A | . | . | A | C | - | - | - | - | - | - | - | - | - | - | - | - | - | - | - | - | - | - | - | - |  |
| Hap_8 | . | C | . | . | G | . | . | . | . | A | . | . | A | C | T | A | T | G | A | A | T | T | G | G | T | C | A | A | A | A | T | T | G | A |  |
| Hap_9 | . | C | . | . | G | . | . | . | T | A | . | . | A | C | - | - | - | - | - | - | - | - | - | - | - | - | - | - | - | - | - | - | - | - |  |
| Hap_10 | . | C | . | . | G | . | . | . | T | A | G | . | A | C | T | A | T | G | A | A | T | T | G | G | T | C | A | A | A | A | T | T | G | A |  |
| Hap_11 | . | . | . | A | G | A | T | G | T | A | G | . | A | C | T | A | T | G | A | A | T | T | G | G | T | C | A | A | A | A | T | T | G | A |  |
| Hap_12 | . | C | . | . | G | . | . | . | T | A | . | . | A | C | T | A | T | G | A | A | T | T | G | G | T | C | A | A | A | A | T | T | G | A |  |
| Hap_13 | . | . | . | . | . | . | . | . | . | A | . | . | . | . | - | - | - | - | - | - | - | - | - | - | - | - | - | - | - | - | - | - | - | - |  |
| Hap_14 | . | C | . | A | G | A | T | G | T | A | . | . | A | C | - | - | - | - | - | - | - | - | - | - | - | - | - | - | - | - | - | - | - | - |  |
| Hap_15 | . | C | . | . | G | . | . | . | . | . | . | . | A | . | T | A | T | G | A | A | T | T | G | G | T | C | A | A | A | A | T | T | G | A |  |
| Hap_16 | . | . | . | . | G | . | . | . | . | . | . | . | A | C | - | - | - | - | - | - | - | - | - | - | - | - | - | - | - | - | - | - | - | - |  |
| Hap_17 | . | C | A | A | G | A | T | G | T | A | . | . | A | C | - | - | - | - | - | - | - | - | - | - | - | - | - | - | - | - | - | - | - | - |  |
| Hap_18 | . | C | . | . | G | . | . | . | . | A | . | . | A | C | - | - | - | - | - | - | - | - | - | - | - | - | - | - | - | - | - | - | - | - |  |
| Hap_19 | . | . | . | . | G | . | . | . | . | A | . | . | A | C | - | - | - | - | - | - | - | - | - | - | - | - | - | - | - | - | - | - | - | - |  |
| Hap_20 | . | C | . | . | G | . | . | . | . | . | . | . | A | C | - | - | - | - | - | - | - | - | - | - | - | - | - | - | - | - | - | - | - | - |  |
| Hap_21 | . | C | . | . | G | . | . | . | T | . | . | . | A | . | T | A | T | G | A | A | T | T | G | G | T | C | A | A | A | A | T | T | G | A |  |
| Hap_22 | . | . | A | A | G | A | T | G | T | A | . | G | A | C | - | - | - | - | - | - | - | - | - | - | - | - | - | - | - | - | - | - | - | - |  |
| Hap_23 | A | C | . | . | G | . | . | . | . | . | . | . | A | . | T | A | T | G | A | A | T | T | G | G | T | C | A | A | A | A | T | T | G | A |  |
| Hap_24 | . | . | . | A | G | A | T | G | T | A | . | G | A | C | - | - | - | - | - | - | - | - | - | - | - | - | - | - | - | - | - | - | - | - |  |
| Hap_25 | . | . | A | . | . | . | . | . | . | . | . | . | . | . | - | - | - | - | - | - | - | - | - | - | - | - | - | - | - | - | - | - | - | - |  |
| Hap_26 | . | . | . | A | G | A | T | G | T | A | . | . | A | C | T | A | T | G | A | A | T | T | G | G | T | C | A | A | A | A | T | T | G | A |  |
| Hap_27 | A | C | . | . | G | . | . | . | . | . | . | . | . | . | - | - | - | - | - | - | - | - | - | - | - | - | - | - | - | - | - | - | - | - |  |
| Hap_28 | . | . | . | . | . | . | . | . | . | . | . | . | A | . | T | A | T | G | A | A | T | T | G | G | T | C | A | A | A | A | T | T | G | A |  |
| Hap_29 | . | C | . | . | G | A | T | G | T | A | . | . | A | C | - | - | - | - | - | - | - | - | - | - | - | - | - | - | - | - | - | - | - | - |  |
| Hap_30 | A | . | . | A | G | A | T | G | T | A | . | . | A | C | - | - | - | - | - | - | - | - | - | - | - | - | - | - | - | - | - | - | - | - |  |
| Hap_31 | . | . | A | A | G | A | T | G | T | A | G | . | A | C | - | - | - | - | - | - | - | - | - | - | - | - | - | - | - | - | - | - | - | - |  |
| Hap_32 | A | C | . | . | G | . | . | . | . | . | . | . | A | C | T | A | T | G | A | A | T | T | G | G | T | C | A | A | A | A | T | T | G | A |  |
| Hap_33 | . | C | . | . | G | . | . | . | . | . | . | . | A | . | - | - | - | - | - | - | - | - | - | - | - | - | - | - | - | - | - | - | - | - |  |
| Hap_34 | . | . | . | . | G | . | . | . | . | . | . | . | A | . | - | - | - | - | - | - | - | - | - | - | - | - | - | - | - | - | - | - | - | - |  |
| Hap_35 | . | C | A | A | G | A | T | G | T | A | . | G | A | C | - | - | - | - | - | - | - | - | - | - | - | - | - | - | - | - | - | - | - | - |  |
| Hap_36 | . | C | . | . | G | A | . | . | T | A | G | . | A | C | T | A | T | G | A | A | T | T | G | G | T | C | A | A | A | A | T | T | G | A |  |
|  |  |  |  |  |  |  |  |  |  |  |  |  |  |  |  |  |  |  |  |  |  |  |  |  |  |  |  |  |  |  |  |  |  |  |  |
| *Lfy* |  |  | 1 | 1 | 1 | 1 | 1 | 1 | 1 | 2 | 2 | 2 | 2 | 2 | 2 | 2 | 3 | 3 | 3 | 3 | 4 | 4 | 4 | 5 | 5 | 5 | 5 | 5 | 6 | 6 | 6 | 6 | 6 | 7 | 7 |
|  | 3 | 4 | 1 | 1 | 1 | 2 | 4 | 5 | 9 | 0 | 2 | 3 | 4 | 5 | 7 | 7 | 1 | 3 | 6 | 7 | 1 | 2 | 6 | 1 | 5 | 6 | 7 | 8 | 0 | 4 | 7 | 8 | 9 | 2 | 2 |
|  | 8 | 5 | 1 | 2 | 5 | 1 | 5 | 4 | 4 | 4 | 3 | 2 | 5 | 2 | 1 | 9 | 7 | 7 | 6 | 8 | 8 | 8 | 2 | 5 | 7 | 8 | 5 | 2 | 2 | 9 | 4 | 0 | 3 | 0 | 1 |
| Hap_1 | A | T | T | - | T | C | T | T | G | A | A | A | T | T | C | A | A | T | G | G | T | G | A | T | G | G | T | A | C | A | T | A | G | G | A |
| Hap_2 | . | . | . | - | . | . | . | . | . | . | . | . | . | . | . | . | . | . | . | . | C | . | . | . | . | . | . | . | . | . | . | . | . | . | . |
| Hap_3 | . | . | . | A | A | T | . | G | . | . | . | . | . | . | . | . | G | . | A | . | . | . | . | A | . | . | . | . | . | . | C | . | . | . | . |
| Hap_4 | . | . | . | A | A | T | . | G | . | . | . | . | . | . | . | . | G | . | A | . | . | . | . | A | . | . | . | . | T | G | . | . | . | . | . |
| Hap_5 | . | . | . | - | . | . | . | G | . | . | . | . | . | . | . | . | . | . | . | T | . | . | . | A | . | . | . | . | . | . | . | G | . | . | . |
| Hap_6 | . | . | . | A | A | T | . | G | . | . | . | . | . | . | . | . | G | C | A | . | . | . | . | A | . | A | . | . | T | G | . | . | . | . | . |
| Hap_7 | . | . | . | - | . | . | . | . | . | . | . | . | . | . | . | . | . | . | A | . | . | . | . | A | . | . | . | . | . | . | C | . | . | . | . |
| Hap_8 | . | . | . | A | A | T | . | G | . | . | . | . | . | . | . | . | G | . | A | . | . | . | . | . | . | . | . | . | . | . | . | . | . | . | . |
| Hap_9 | . | C | . | - | . | . | C | G | . | . | . | . | C | . | . | . | . | . | . | T | . | . | . | A | A | . | C | . | . | . | C | . | . | . | . |
| Hap_10 | . | . | . | - | . | . | . | G | . | . | . | . | . | . | . | . | . | . | . | T | . | A | . | A | . | . | . | . | . | . | . | G | . | . | . |
| Hap_11 | . | . | . | - | . | . | . | G | . | . | . | . | . | . | . | . | . | . | . | . | . | . | . | A | . | . | . | . | . | . | . | . | . | . | . |
| Hap_12 | . | . | . | - | . | . | . | G | . | . | . | . | . | . | . | . | . | . | . | . | . | . | . | A | . | . | . | T | . | . | . | . | . | . | . |
| Hap_13 | . | . | . | - | . | . | . | . | . | . | . | . | . | . | . | T | . | . | . | . | . | . | . | . | . | . | . | . | . | . | . | . | . | . | G |
| Hap_14 | . | . | . | - | . | . | . | G | . | . | . | . | . | . | . | . | . | . | . | . | C | . | . | A | . | . | . | . | . | . | . | . | . | . | . |
| Hap_15 | . | . | . | - | . | . | . | G | . | . | . | . | . | . | . | . | . | . | . | T | . | . | . | A | . | . | . | . | . | . | C | . | . | . | . |
| Hap_16 | . | . | . | A | A | T | . | G | A | . | . | G | . | . | . | . | G | . | A | . | . | . | . | A | . | . | . | . | T | G | . | . | . | . | . |
| Hap_17 | . | . | . | - | . | . | . | G | . | . | . | . | . | . | . | . | . | . | . | . | . | . | . | . | . | . | . | . | . | . | . | . | . | . | . |
| Hap_18 | . | . | . | A | A | T | . | G | . | . | . | . | . | . | . | . | G | . | A | . | . | . | . | A | . | . | . | . | T | . | . | . | . | . | . |
| Hap_19 | . | . | . | - | . | . | . | G | . | . | . | . | . | . | . | . | . | . | . | . | . | . | . | A | . | . | . | . | . | . | . | G | . | . | . |
| Hap_20 | . | . | . | A | A | T | . | G | . | . | . | . | . | . | T | . | . | . | . | T | . | . | G | A | . | . | . | . | . | . | . | G | . | . | . |
| Hap_21 | G | . | . | - | . | . | . | G | . | . | . | . | . | . | . | . | . | . | . | T | . | . | . | A | . | . | . | . | . | . | . | G | . | A | . |
| Hap_22 | . | . | . | - | . | . | . | . | . | . | . | . | . | . | . | . | . | . | . | . | . | . | . | . | . | . | . | . | . | . | . | . | . | A | . |
| Hap_23 | . | . | C | A | A | T | . | G | . | . | . | . | . | C | . | . | G | . | A | . | . | . | . | A | . | . | . | . | T | G | . | . | . | . | . |
| Hap_24 | . | . | . | - | . | . | . | . | . | . | . | . | . | . | . | . | . | . | . | . | C | . | . | . | . | . | . | . | . | . | . | . | . | A | . |
| Hap_25 | . | . | . | - | . | . | . | . | . | . | . | . | . | . | . | . | . | . | . | . | C | . | . | A | . | . | . | . | . | . | . | . | . | . | . |
| Hap_26 | . | . | . | - | . | . | . | . | . | . | . | . | . | . | . | . | . | . | . | . | . | . | . | A | . | . | . | . | . | . | . | . | . | . | . |
| Hap_27 | . | . | . | A | A | T | . | G | . | . | . | . | . | . | . | . | . | . | . | . | C | . | . | A | . | . | . | . | . | . | . | . | . | . | . |
| Hap_28 | . | . | . | - | . | . | . | . | . | . | . | . | . | . | . | . | . | . | . | T | . | . | . | A | . | . | . | . | . | . | . | . | . | . | . |
| Hap_29 | . | . | . | - | . | . | . | . | . | . | . | . | . | . | . | . | . | . | . | T | . | . | . | . | . | . | . | . | . | . | . | G | . | . | . |
| Hap_30 | . | . | . | - | . | . | . | . | . | . | . | . | . | . | . | . | . | . | . | . | . | . | . | A | . | . | . | . | . | . | . | G | . | . | . |
| Hap_31 | . | . | . | - | . | . | . | G | . | . | . | . | . | . | . | . | . | . | . | . | . | . | . | . | . | . | . | . | . | . | . | G | . | . | . |
| Hap_32 | . | . | . | A | A | T | . | G | . | . | . | . | . | . | . | . | G | . | . | . | . | . | . | A | . | . | . | . | T | . | . | . | . | . | . |
| Hap_33 | . | . | . | - | . | . | . | G | . | T | G | . | . | . | . | . | . | . | . | T | . | . | . | A | . | . | . | . | . | . | . | G | C | . | . |
| Hap_34 | G | . | . | - | . | . | . | G | . | . | . | . | . | . | . | . | . | . | . | . | . | . | . | A | . | . | . | T | . | . | . | . | . | . | . |
| Hap_35 | . | . | . | - | . | . | . | G | . | . | . | . | . | . | . | . | . | . | . | . | . | . | . | . | . | . | . | . | . | . | C | . | . | . | . |

The shaded regions of the polymorphic sites indicate regions that were removed from genealogical-based analyses in consideration of intralocus recombination.
