# Supplementary material for: Historical spatial range expansion and a very recent bottleneck of Cinnamomum kanehirae Hay. (Lauraceae) in Taiwan inferred from nuclear genes
Source: BMC Evol Biol. 2010 Apr 30;10:124. doi: 10.1186/1471-2148-10-124 (PMC2880300; doi:10.1186/1471-2148-10-124)
Supplement: Additional file 2 — Pairwise FST of 19 Cinnamomum kanehirae populations. The table provides the pairwise genetic distance matrix obtained using Chs and Lfy DNA. [file 1471-2148-10-124-S2.DOC]

**Table S2:** ***Pairwise F_ST_ of 19 Cinnamomum kanehirae populations.***

|  | ALS | CL | CP | CT | FKS | FL | HKL | HNS | KS | KW | NC | TH | TML | TN | TP | WC | WH | YF | YL |
| --- | --- | --- | --- | --- | --- | --- | --- | --- | --- | --- | --- | --- | --- | --- | --- | --- | --- | --- | --- |
| ALS | - | 0.489 | 0.418 | 0.526 | 0.051 | 0.226 | 0.410 | 0.000 | 0.129 | 0.238 | 0.182 | 0.182 | 0.485 | 0.222 | 0.273 | 0.387 | 0.333 | 0.000 | 0.414 |
| CL | 0.069 | - | 0.192 | 0.278 | 0.201 | 0.143 | 0.137 | 0.256 | 0.104 | 0.306 | 0.278 | 0.278 | 0.338 | 0.040 | 0.139 | 0.038 | 0.410 | 0.017 | 0.159 |
| CP | 0.018 | 0.102 | - | 0.249 | 0.170 | 0.075 | 0.033 | 0.199 | 0.099 | 0.249 | 0.227 | 0.227 | 0.291 | 0.077 | 0.075 | 0.017 | 0.343 | 0.010 | 0.066 |
| CT | 0.042 | 0.068 | 0.058 | - | 0.132 | 0.148 | 0.134 | 0.000 | 0.124 | 0.000 | 0.125 | 0.125 | 0.410 | 0.200 | 0.135 | 0.108 | 0.308 | 0.133 | 0.071 |
| FKS | 0.004 | 0.165 | 0.210 | 0.061 | - | 0.010 | 0.110 | 0.186 | 0.038 | 0.068 | 0.100 | 0.100 | 0.199 | 0.029 | 0.022 | 0.090 | 0.098 | 0.139 | 0.041 |
| FL | 0.066 | 0.087 | 0.016 | 0.058 | 0.150 | - | 0.060 | 0.000 | 0.052 | 0.076 | 0.055 | 0.055 | 0.214 | 0.071 | 0.160 | 0.093 | 0.196 | 0.209 | 0.156 |
| HKL | 0.032 | 0.064 | 0.029 | 0.034 | 0.169 | 0.014 | - | 0.087 | 0.064 | 0.174 | 0.134 | 0.134 | 0.188 | 0.009 | 0.089 | 0.044 | 0.329 | 0.023 | 0.155 |
| HNS | 0.133 | 0.454 | 0.352 | 0.133 | 0.310 | 0.012 | 0.358 | - | 0.103 | 0.333 | 0.364 | 0.364 | 0.121 | 0.098 | 0.020 | 0.074 | 0.000 | 0.160 | 0.000 |
| KS | 0.051 | 0.055 | 0.032 | 0.048 | 0.177 | 0.037 | 0.049 | 0.425 | - | 0.007 | 0.029 | 0.029 | 0.232 | 0.020 | 0.022 | 0.059 | 0.069 | 0.126 | 0.010 |
| KW | 0.088 | 0.180 | 0.018 | 0.034 | 0.134 | 0.143 | 0.078 | 0.024 | 0.117 | - | 0.375 | 0.375 | 0.300 | 0.109 | 0.078 | 0.160 | 0.067 | 0.042 | 0.050 |
| NC | 0.073 | 0.035 | 0.037 | 0.046 | 0.079 | 0.101 | 0.023 | 0.171 | 0.012 | 0.086 | - | 0.500 | 0.258 | 0.077 | 0.063 | 0.108 | 0.000 | 0.054 | 0.000 |
| TH | 0.024 | 0.179 | 0.026 | 0.053 | 0.178 | 0.022 | 0.085 | 0.272 | 0.100 | 0.083 | 0.044 | - | 0.258 | 0.077 | 0.063 | 0.108 | 0.000 | 0.054 | 0.000 |
| TML | 0.013 | 0.100 | 0.032 | 0.013 | 0.139 | 0.018 | 0.084 | 0.342 | 0.071 | 0.072 | 0.037 | 0.082 | - | 0.254 | 0.174 | 0.317 | 0.485 | 0.136 | 0.291 |
| TN | 0.089 | 0.344 | 0.290 | 0.047 | 0.218 | 0.004 | 0.258 | 0.122 | 0.311 | 0.035 | 0.101 | 0.233 | 0.233 | - | 0.022 | 0.034 | 0.255 | 0.131 | 0.013 |
| TP | 0.082 | 0.065 | 0.049 | 0.058 | 0.026 | 0.073 | 0.025 | 0.117 | 0.045 | 0.072 | 0.070 | 0.018 | 0.001 | 0.031 | - | 0.088 | 0.184 | 0.178 | 0.182 |
| WC | 0.039 | 0.105 | 0.012 | 0.070 | 0.178 | 0.054 | 0.068 | 0.441 | 0.059 | 0.117 | 0.010 | 0.073 | 0.084 | 0.355 | 0.060 | - | 0.345 | 0.102 | 0.250 |
| WH | 0.059 | 0.002 | 0.088 | 0.078 | 0.060 | 0.126 | 0.046 | 0.080 | 0.002 | 0.040 | 0.078 | 0.039 | 0.080 | 0.026 | 0.106 | 0.048 | - | 0.080 | 0.261 |
| YF | 0.025 | 0.074 | 0.005 | 0.033 | 0.173 | 0.018 | 0.078 | 0.368 | 0.068 | 0.068 | 0.041 | 0.050 | 0.095 | 0.272 | 0.026 | 0.110 | 0.029 | - | 0.133 |
| YL | 0.078 | 0.103 | 0.053 | 0.079 | 0.187 | 0.118 | 0.038 | 0.504 | 0.055 | 0.183 | 0.024 | 0.140 | 0.065 | 0.391 | 0.086 | 0.126 | 0.081 | 0.073 | - |

Above the diagonal: estimates from *Lfy*.

Below the diagonal: estimates from *Chs*.

Sampling locations are described in Table 1.
