# Supplementary material for: Historical spatial range expansion and a very recent bottleneck of Cinnamomum kanehirae Hay. (Lauraceae) in Taiwan inferred from nuclear genes
Source: BMC Evol Biol. 2010 Apr 30;10:124. doi: 10.1186/1471-2148-10-124 (PMC2880300; doi:10.1186/1471-2148-10-124)
Supplement: Additional file 3 — Phylogenetic network constructed by both Chs and Lfy sequences. The figure shows the haplotype networks constructed using a statistical parsimony approach. The long-fragment indel of Chs sequences was treated as one-mutation step and recoded. The limit of 90% connection was used to estimate the network. Four geographic regions were indicated proportionally with four colors. [file 1471-2148-10-124-S3.DOC]

Additional file 3


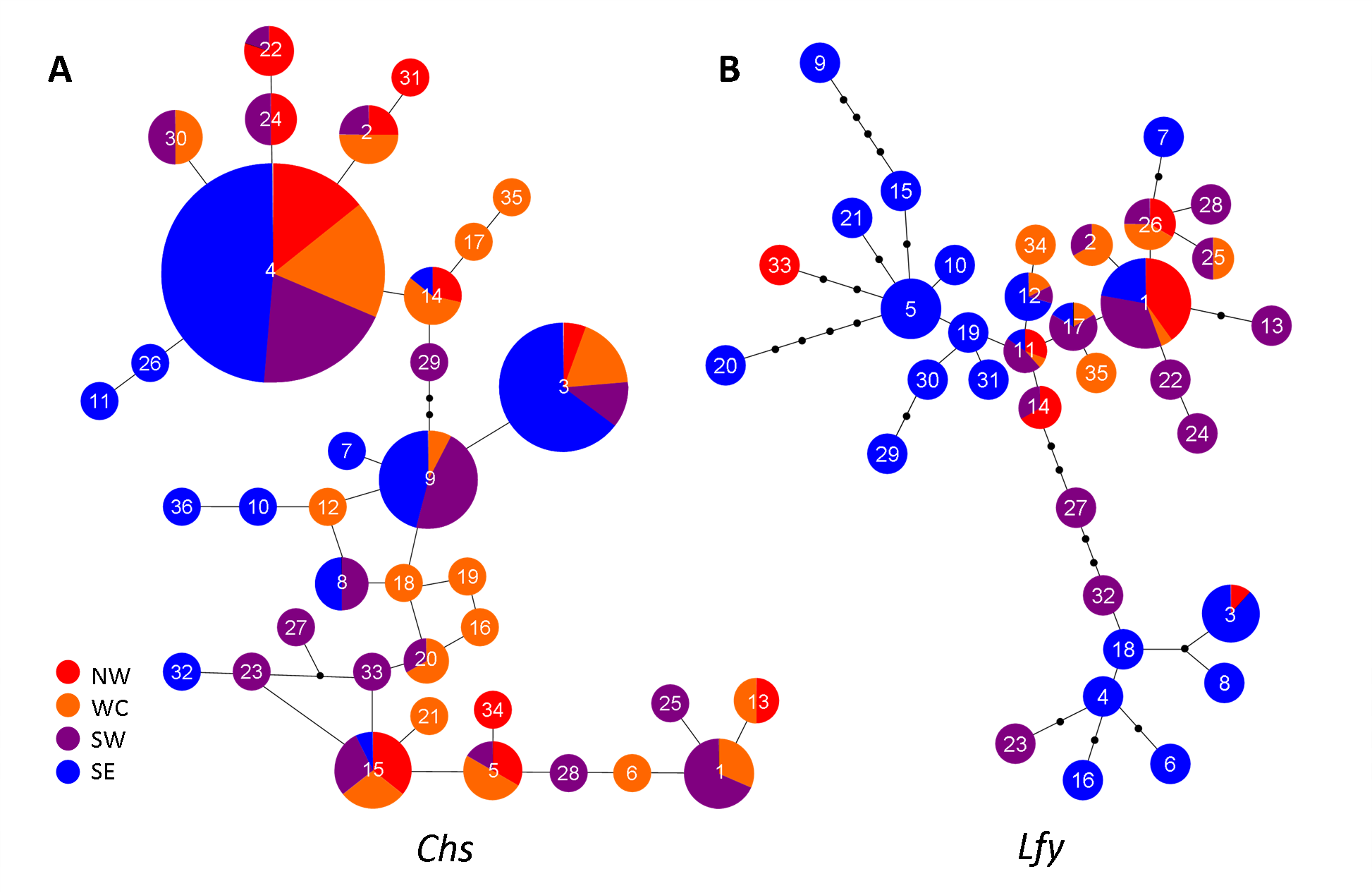


***Phylogenetic network constructed by both* Chs *and* Lfy *sequences.*** *The figure shows the haplotype networks constructed using a statistical parsimony approach. The long-fragment indel of* Chs *sequences was treated as one-mutation step and recoded. The limit of 90% connection was used to estimate the network. Four geographic regions were indicated proportionally with four colors.*
