# Supplementary figures and images for: Historical spatial range expansion and a very recent bottleneck of Cinnamomum kanehirae Hay. (Lauraceae) in Taiwan inferred from nuclear genes
Source: BMC Evol Biol. 2010 Apr 30;10:124. doi: 10.1186/1471-2148-10-124 (PMC2880300; doi:10.1186/1471-2148-10-124)

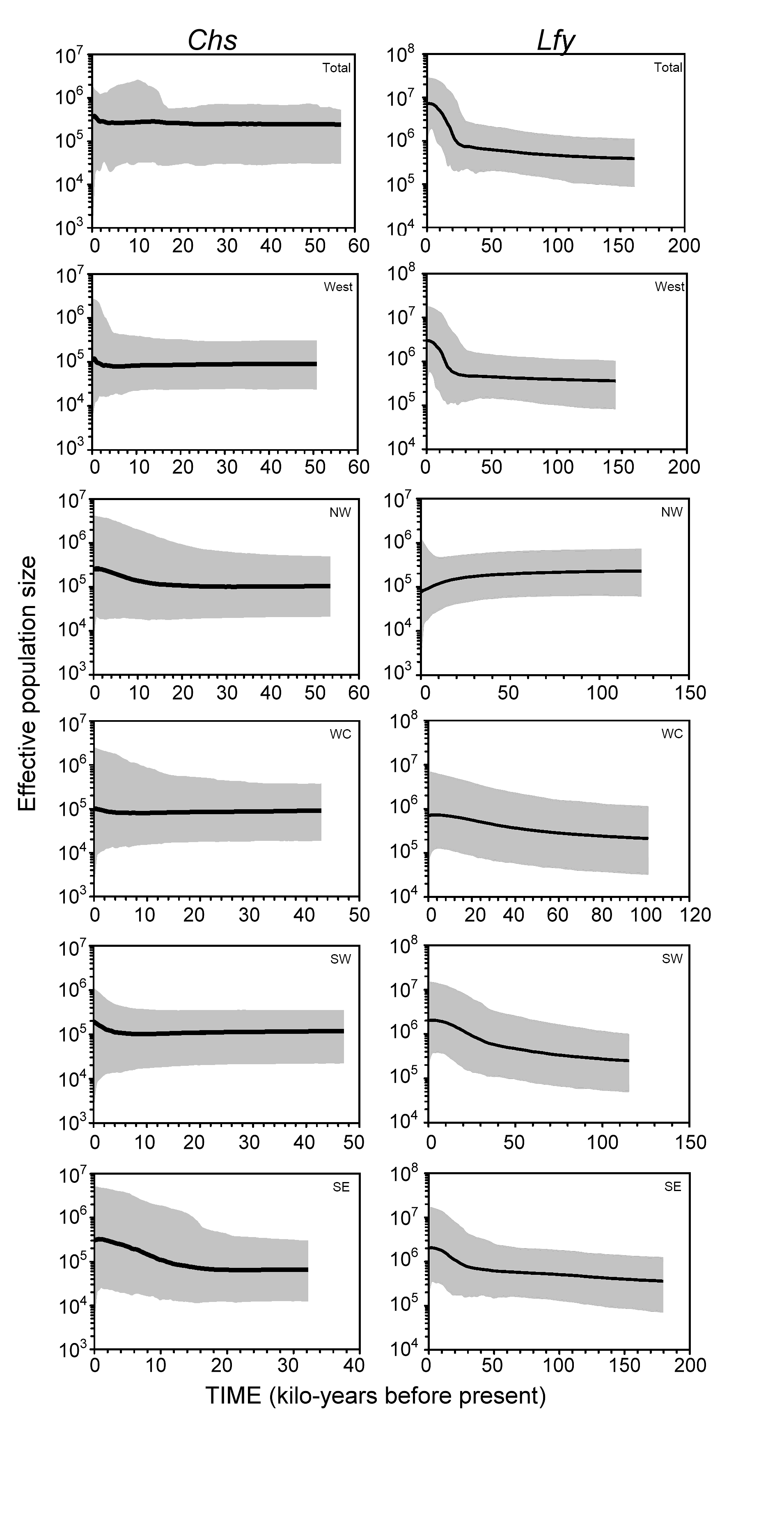

Supplement: Additional file 4 — Changes in effective population size in regional and pooled samples of Cinnamomum kanehirae. The figure shows the effective population size per generation for regional and pooled samples of Cinnamomum kanehirae based on Chs and Lfy datasets. The solid line represents mean values of the estimated effective population size with lower (0.05) and upper (0.95) bounds. [file 1471-2148-10-124-S4.DOC]
